# Enhancing Resistive Switching in AlN-Based Memristors Through Oxidative Al_2_O_3_ Layer Formation: A Study on Preparation Techniques and Performance Impact

**DOI:** 10.3390/mi15121499

**Published:** 2024-12-16

**Authors:** Hongxuan Guo, Jiahao Yao, Siyuan Chen, Chong Qian, Xiangyu Pan, Kuibo Yin, Hao Zhu, Xu Gao, Suidong Wang, Litao Sun

**Affiliations:** 1 School of Integrated Circuit, Southeast University, Nanjing 210096, China; yaojiahao@seu.edu.cn (J.Y.); chensiyuanshu@163.com (S.C.); yinkuibo@seu.edu.cn (K.Y.); slt@seu.edu.cn (L.S.); 2 Institute of Functional Nano & Soft Materials (FUNSOM), Soochow University, Suzhou 215123, China; qc19991014@163.com (C.Q.); 20244014058@stu.suda.edu.cn (X.P.); gaoxu@suda.edu.cn (X.G.);; 3 School of Microelectronics, Fudan University, Shanghai 200433, China; hao_zhu@fudan.edu.cn

**Keywords:** aluminum nitride (AlN), sputtering, aluminum oxide (Al_2_O_3_), memristor, resistive random access memory (RRAM), conductive filaments (CFs)

## Abstract

Aluminum nitride (AlN) with a wide band gap (approximately 6.2 eV) has attractive characteristics, including high thermal conductivity, a high dielectric constant, and good insulating properties, which are suitable for the field of resistive random access memory. AlN thin films were deposited on ITO substrate using the radio-frequency magnetron sputtering technique. Al’s and Au’s top electrodes were deposited on AlN thin films to make a Au/Al/AlN/ITO sandwich structure memristor. The effects of the Al_2_O_3_ film on the on/off window and voltage characteristics of the device were investigated. The deposition time and nitrogen content in the sputtering atmosphere were changed to adjust the thickness and composition of AlN films, respectively. The possible mechanism of resistive switching was examined via analyses of the electrical resistive switching characteristics, forming voltage, and switching ratio.

## 1. Introduction

With the rapid development of big data and artificial intelligence technologies, modern computing systems are facing significant challenges in data processing capabilities. The traditional von Neumann architecture suffers from the “von Neumann bottleneck” caused by the disparity in data transfer speed between memory and processors, which limits the efficiency and speed of computation. This bottleneck has driven the exploration of novel memory technologies to overcome the limitations of conventional architectures. The memristor, an emerging type of non-volatile memory, has attracted significant attention due to its advantages, such as high-speed operation, low power consumption, high integration density, and synaptic-like behavior. These features make memristors highly promising for applications in storage, logic operations, machine learning, and neuromorphic computing [1,2,3].

The memristor’s resistive switching capability enables it to transition between high-resistance states (HRSs) and low-resistance states (LRSs) under applied voltage, which not only allows for data storage but also serves as the basic computational unit for artificial neural networks. This unique feature makes it a core component in neuromorphic computing [4]. To date, research on memristor material systems spans binary metal oxides [5,6,7,8], perovskite complex oxides [9,10], solid electrolytes [11,12], organic polymers [13,14], elemental semiconductors [15,16], and nitride materials [17,18]. Among these, nitride-based materials have garnered increasing attention due to their excellent properties. In recent years, nitride-based memristors have attracted growing interest due to their excellent material properties, such as high thermal conductivity, high dielectric constant, and wide bandgap, making them promising candidates for non-volatile memory devices. Although several studies have explored nitride-based memristors, their development remains in the early stages compared to other material systems. Most research has focused on widely studied materials such as binary metal oxides. In this context, the present study builds upon previous work in the field of nitride-based memristors, aiming to investigate the potential of aluminum nitride (AlN) films for resistive switching applications. AlN offers several unique advantages, including better processing compatibility with CMOS technology and promising resistive switching behavior [19]. This work explores the fabrication and characterization of AlN-based memristors, with the goal of enhancing device performance and uncovering the underlying mechanisms that could lead to their broader application in memory devices and logic circuits.

Recent studies have shown that introducing a thin aluminum oxide (Al_2_O_3_) layer on the surface of AlN films can significantly improve the performance of memristors. The Al_2_O_3_ layer not only provides additional oxygen vacancies to stabilize conductive pathways but also expands the switching window, thereby enhancing the device’s performance in both storage and computing applications [20,21]. Based on this finding, the present study employs radio-frequency magnetron sputtering to fabricate both AlN films and Al_2_O_3_/AlN composite structures, systematically investigating the resistive switching behaviors of these two structures. By analyzing typical I-V characteristics and the formation mechanisms of conductive filaments, the critical roles of nitrogen and oxygen vacancies in the switching process are elucidated.

Furthermore, this work demonstrates the improvements in device consistency and stability achieved through oxidation treatment, providing both theoretical insights and experimental evidence for the future design of high-performance memristors. Through an in-depth exploration of the resistive switching mechanisms of nitride-based memristors and the synergistic effects of composite structures, this study lays the theoretical foundation for the broader application of nitride-based memristors and provides important references for the development of new, high-performance storage and computing devices.

## 2. Experimental Procedure

AlN films were grown on a 2 cm × 2 cm ITO substrate using an RF magnetron sputtering system (NANO36, the FUNSOM experimental platform of Soochow University) from a pure Al (99.999%) target at room temperature. The sputtering conditions of the AlN films were as follows: At a flow rate of Ar = 40 sccm and N_2_ = 10 sccm in the chamber, the aluminum target was sputtered with the mixed gas at a power of 250 W, and the working pressure was about 5 mTorr. The targets for top electrode sputtering were pure Al (99.999%) and pure Au (99.999%). Detailed conditions of the sputtering process are listed in Table 1. The thickness of AlN films under different working gases and different deposition times was determined using an ellipsometer. I-V measurements were performed using a semiconductor parameter analysis system (Keithley 4200, the FUNSOM experimental platform of Soochow University) to confirm resistive switching. The top electrodes were circular with diameters of 400 μm and 200 μm, deposited from a stainless steel mask. The effect on the device’s on/off window and voltage characteristics were analyzed by comparing whether the Al_2_O_3_ films were generated by oxidation on the top of the AlN films.

## 3. Results and Discussion

The resistive switching characteristics of AlN-based memristors were investigated. An Au/Al/AlN/ITO device based on an ITO substrate was fabricated to verify the function of an AlN-based memristor. AlN thin films were grown via magnetron sputtering, and the surfaces of the AlN thin films were oxidized to form a thin Al_2_O_3_ layer by providing oxygen. The bottom electrode was ITO, the top electrode was Au, and the metal Al was mainly used as a buffer layer for adhesion.

**Figure 1:**
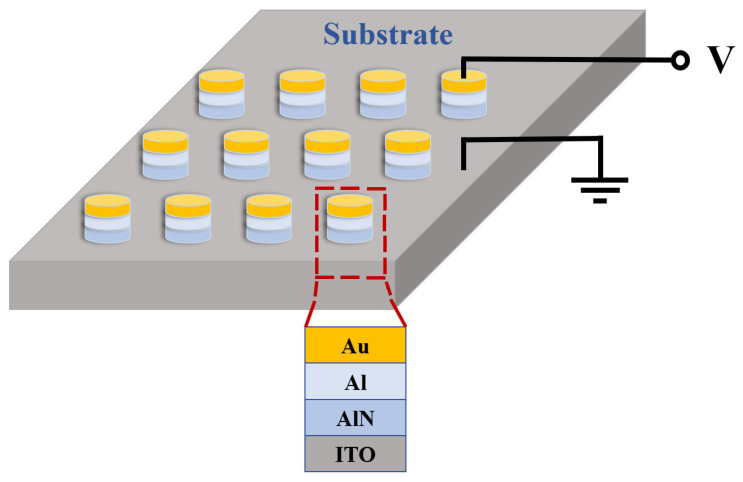
Device structure of Au/Al/AlN/ITO memory cell.

**Figure 2:**
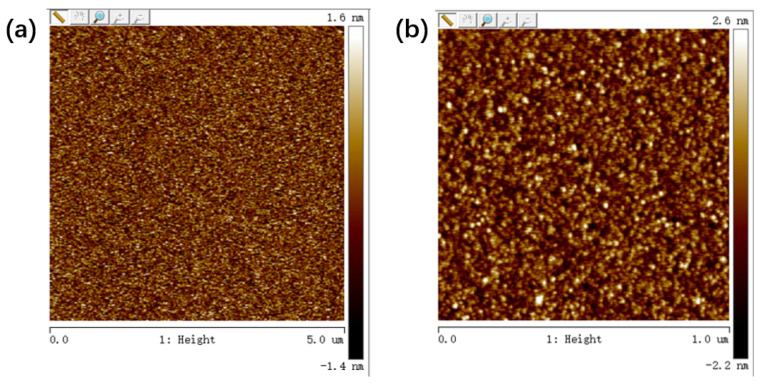
AFM images of AlN films with areas of (**a**) 5 × 5 µm^2^ and (**b**) 1 × 1 µm^2^. Their root mean square roughnesses are 0.430 nm and 0.677 nm, respectively.

**Figure 3:**
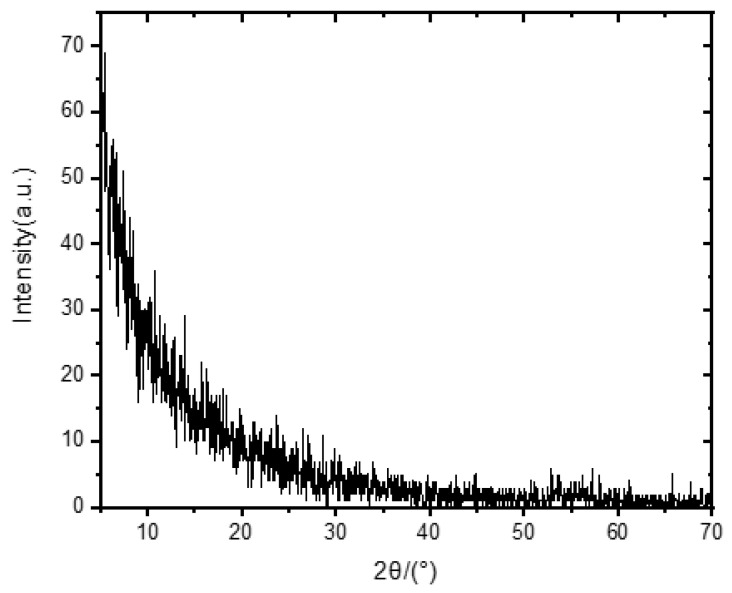
XRD results of AlN thin films on a silicon substrate.

Figure 1 shows the schematic of the Au/Al/AlN/ITO memory cell. The surface morphology of the AlN film deposited on the silicon substrate was observed using atomic force microscopy (AFM) shown in Figure 2a,b. The AFM micrograph shows that the root mean square roughness of AlN films over 5 × 5 µm^2^ and 1 × 1 µm^2^ is 0.430 nm and 0.677 nm, respectively. Figure 3 shows the X-ray diffraction pattern of the aluminum nitride film, indicating that the aluminum nitride film is not a crystal structure.

**Figure 4:**
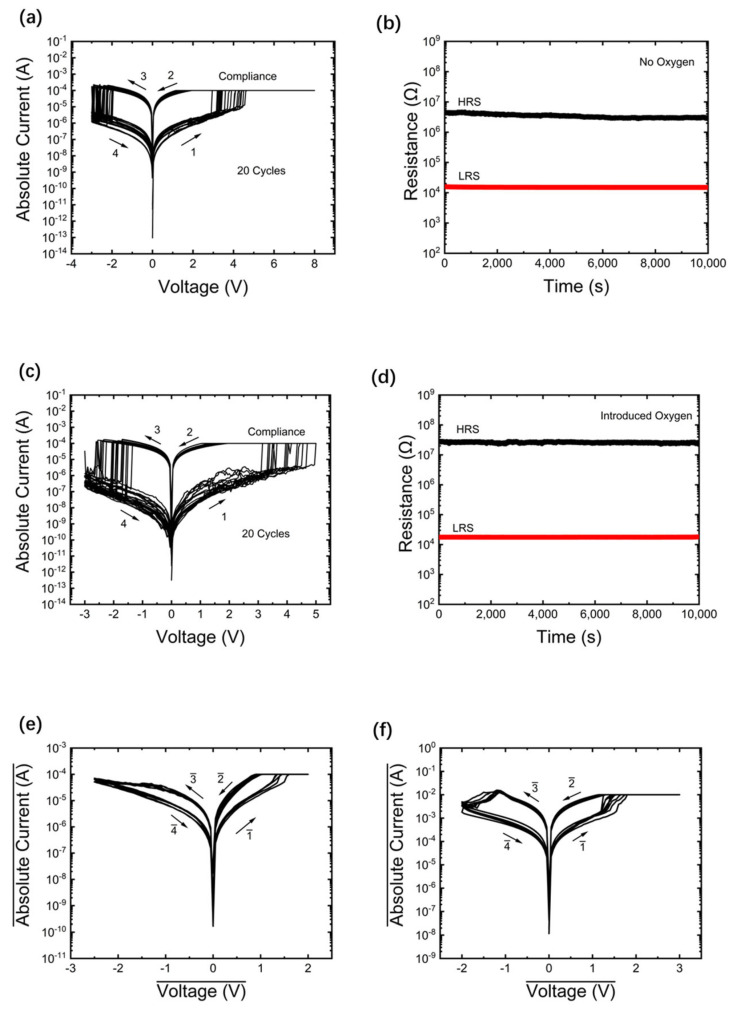
(**a**) Typical I–V 20 cycles of the Au/Al/AlN/ITO device. (**b**) Data retention features of the Au/Al/AlN/ITO device at 0.1 V for 10^4^ s. (**c**) Typical I–V 20 cycles of the Au/Al/Al_2_O_3_/AlN/ITO device. (**d**) Data retention features of the Au/Al/Al_2_O_3_/AlN/ITO device at 0.1 V for 10^4^ s. (**e**) The typical I-V of AlN with a 30 min RF magnetron sputtering time and no oxygen gas and (**f**) the typical I-V of AlN with a 30 min RF magnetron sputtering time and oxygen gas.

So far, several studies have explored the switching mechanisms of AlN-based resistive switching materials, most of which indicate that the resistive switching in AlN-based memristors may be attributed to nitrogen vacancies and/or aluminum vacancies [22,23,24,25]. When the memristor transitions to a low-resistance state (LRS), conductive filaments composed of nitrogen vacancies and/or aluminum vacancies may form within the AlN layer. For memory resistors used in neuromorphic computing systems, low fluctuation is crucial for accurate recognition. Figure 4a illustrates the electroforming process of this device. Notably, our device exhibits excellent consistency in the bipolar resistive switching (BRS) curve. The set voltage is approximately 4 V, and the reset voltage is around −2.5 V. The retention test shown in Figure 4b follows the conventional method for non-volatile memory technologies. First, the device is set to the high-resistance state (HRS) and LRS through SET and RESET operations, respectively. Then, the resistance values of HRS and LRS are measured every 2.5 s using a 0.1 V reading voltage. After 10^4^ s, no significant degradation in the HRS and LRS resistance is observed. After the measurement, the memristor demonstrates excellent non-volatility. Additionally, as shown in Figure 4b, the switching window of the device is approximately 2 × 10^2^.

Next, a new device was fabricated under the same conditions. The only difference was that after the growth of the AlN film, 5 min of oxygen was introduced into the sputtering chamber, resulting in the oxidation of the AlN film surface to form a thin Al_2_O_3_ layer. Al and Au’s top electrodes were then deposited via magnetron sputtering. Similarly, an electroforming process, as shown in Figure 4c, was performed to activate the device and enable subsequent resistive switching characteristics. Specifically, the forming voltage for the Au/Al/Al_2_O_3_/AlN/ITO device is approximately 5.0 V. Figure 4c shows the BRS curve for the Au/Al/Al_2_O_3_/AlN/ITO device. The device is initially in a high-resistance state. By gradually increasing the positive voltage applied to the device, the resistance of the memristor abruptly drops, transitioning from HRS to LRS. When the applied voltage is swept from zero to negative, a “reset” process occurs. When the bias voltage is swept back to zero, the memristor remains in HRS. In this case, the voltage sweep in the BRS curve follows the pattern: 0 V → 5 V → 0 V → −3 V → 0 V.

It is noteworthy that our new device exhibits excellent consistency in the BRS curve. The set voltage is approximately 4 V, and the reset voltage is about −2.0 V. The retention test shown in Figure 4d follows the standard procedure for non-volatile memory technology. The memristor is first switched to either LRS or HRS, and its resistance is repeatedly measured by applying a 0.1 V reading voltage at room temperature. After the measurement, the memristor also demonstrates excellent non-volatility. According to Figure 4d, the switching window of the device is approximately 2 × 10^3^. It can be observed that the device increases the switching window of the memristor without altering its cycling characteristics, thereby significantly enhancing the performance of the memristor.

Furthermore, we performed both oxygen and non-oxygen tests under the condition of AlN magnetron sputtering for 30 min. Figure 4e and Figure 4f display the I–V curves under non-oxygen and oxygen conditions, respectively. From the figures, it is evident that the device’s performance is superior under oxygen conditions compared to non-oxygen conditions. However, the device’s performance under non-oxygen conditions is poorer compared to the device with 5 min of oxygen exposure. Therefore, the subsequent discussion will focus on the device with 5 min of oxygen exposure.

**Figure 5:**
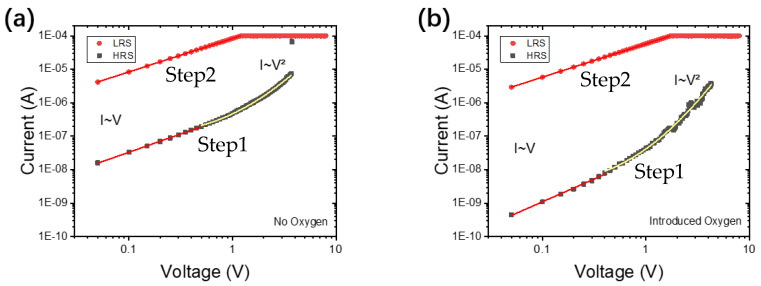
logI–logV plot of the HRS and LRS at positive bias. (**a**) The Au/Al/AlN/ITO device and (**b**) the Au/Al/Al_2_O_3_/AlN/ITO device.

As shown in Figure 5, the resistance switching mechanism is studied by analyzing the current transmission in step 1 and step 2 of the double logarithmic graph of the typical I-V curve. As shown in Figure 5a,b, during the voltage scanning in step 1, it is observed that the current voltage is in a linear ohmic relationship at low to a medium bias voltage (<0.5 V), that is, I∝V. Then, when the voltage sweep is close to 3.0 V, the current voltage relationship is quadratic, that is, I∝V^2^. A sharp increase in current was observed at 3.5 V (VSET), and then the secondary current voltage behavior reappeared until the current was clamped by the compliance setting. Trap-related space charge limiting current (SCLC) has aptly described this current transport mechanism, and VSET corresponds to the voltage at which all traps in AlN are filled with injected carriers. Therefore, we believe that the resistance switching mechanism in our devices is due to the trap-related SCLC mechanism. In step 1 of VSET, the complete filling of these traps causes the device resistance to change from HRS to LRS, while in step 2, the release of these traps causes the device resistance to change from LRS to HRS. From the results of material analysis, AlN film is the provider of nitrogen ions, and this process will leave a large number of nitrogen vacancies in AlN film. These point defects in AlN film may play the role of trap center, which leads to bipolar resistance switching of devices through the SCLC mechanism.

**Figure 6:**
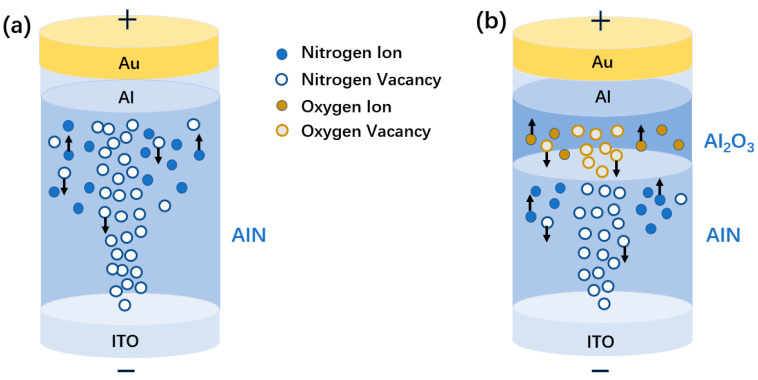
Schematic diagram of the bipolar IV switch process of (**a**) the Au/Al/AlN/ITO device and (**b**) the Au/Al/Al_2_O_3_/AlN/ITO device.

It is generally believed that the electroresistive effect of memristors is dominated by the formation and fracture of conductive filaments in the resistive functional layer. Since here is no active electrode in the Au/Al/AlN/ITO device, the possibility of active electrode metal forming conductive filament is ruled out, and its resistance transition behavior is mainly dominated by the N-vacancy conductive filament mechanism. Figure 6 shows the schematic diagram of the bipolar IV switch process of AlN film. When enough positive bias voltage is applied on the Au electrode, the aluminum nitrogen covalent bond in aluminum nitride breaks to form ions. Under the action of the electric field, N ions move toward the AlN/Al interface, and N vacancies are generated in the material, forming N vacancy conductive filaments. The device changes from an HRS to an LRS. Under the opposite bias voltage, N ions at the AlN/Al interface migrate to the direction of the ITO electrode, partially filling the N vacancy, making the conductive filament partially disconnected, and the device changes from LRS to HRS. In general, boron nitride films grown by magnetron sputtering contain many defects (such as vacancies, grain boundaries, etc.), which contribute to the resistance transition behavior of devices so that AlN-based devices can use the electrical activation process to induce the subsequent resistance transition behavior [26].

For devices fabricated by oxidizing AlN films with oxygen to form Al_2_O_3_ layers, the resistance transition behavior is mainly controlled by the conductive wire mechanism consisting of N-vacancy and O-vacancy. When sufficient positive bias voltage is applied to the Au electrode, the aluminum nitrogen covalent bond in aluminum nitride breaks, and the aluminum oxygen bond in aluminum oxide breaks to form ions. Under the action of the electric field, N ions and O ions move toward the Au electrode, resulting in N vacancies and O vacancies in the material, forming N and O vacancy conducting wires. The device changes from HRS to LRS. Under the opposite bias voltage, N ions and O ions on the interface migrate to the ITO electrode, partially filling N and O vacancies so that the conductive wire is partially disconnected, and the device is transformed from LRS to HRS.

Table 2 compares the devices in our study with those from other studies in the literature, focusing on three key performance metrics: the switching ratio, cycle endurance, and retention time. Our device demonstrates good performance across these indicators. It is important to note that the core of this study is exploring the impact of oxygen on device performance, and the introduction of aluminum oxide (Al_2_O_3_) into the device has significantly improved its performance. As shown in Table 2, the addition of aluminum oxide has a positive effect on the device’s performance, further validating the crucial role of oxygen in optimizing device performance.

## 4. Conclusions

This work provides insights into the resistive switching mechanisms and performance enhancements of AlN-based and Al_2_O_3_/AlN composite memristor devices. In AlN-based devices, resistive switching is governed by the formation and rupture of nitrogen vacancy (N-vacancy) conductive filaments, transitioning between high-resistance states (HRSs) and low-resistance states (LRSs). The introduction of an Al_2_O_3_ layer significantly improves device performance by stabilizing conductive pathways through the synergistic effect of nitrogen and oxygen vacancies, resulting in an expanded switching window, enhanced stability, and improved consistency. The resistive switching behavior is attributed to a trap-controlled space charge limited current (SCLC) mechanism. Retention tests confirm excellent non-volatility, with stable resistance states over $10^{4}$ seconds. These findings highlight the potential of Al_2_O_3_/AlN composite structures for non-volatile memory and neuromorphic computing, with further optimization of interfaces and oxidation methods offering opportunities for performance improvement.

## Figures and Tables

**Table 1:** Detail conditions of the sputtering process.

	Condition of AlN Films Deposition	Condition of Al Top Electrode	Condition of Au Top Electrode
Target	Al (99.999%)	Al (99.999%)	Au (99.999%)
Substrate temperature (°C)	No heating	No heating	No heating
Base pressure (Torr)	<8.0 $\times \;10^{-6}$	<8.0 $\times \;10^{-6}$	<8.0 $\times \;10^{-6}$
Working pressure (mTorr)	5.0	5.0	5.0
RF power (Watt)	AC250	DC50	DC100
Ar flow/N_2_ flow	40 sccm/10 sccm	43 sccm/0 sccm	43 sccm/0 sccm

**Table 2:** Memristor material performance comparison.

Model	Switching Ratio	Cycle	Retention Time (s)
Al/Ta_2_O_5_/ITO [27]	~10^2^	>50	~10^2^
ITO/Ta_2_O_5_/ITO [27]	~10	>50	~10^2^
Al/ZnO/ITO [28]	10^4^	100	10^4^
Pt/aTa_2_O_5_/TiN [29]	10^2^	None	10^4^
Au/Al/AlN/ITO (this work)	~10^2^	100	10^4^
Au/Al/Al_2_O_3_/AlN/ITO (this work)	10^3^	>20	10^4^

## Data Availability

The data presented in this study are available on request from the corresponding author.
